# Recombinant Cell-Permeable Puromycin *N*-Acetyltransferase Confers Puromycin Resistance by Direct Protein Transduction

**DOI:** 10.4014/jmb.2502.02049

**Published:** 2025-07-18

**Authors:** Jiwon Choi, Kyung-Hee Cho, Jiwon Im, Yeeun Seo, Amitesh Sharma, Kartic, Shivani Devi, Nattan Stalin, Seo Jin Park, Tae-Sik Park

**Affiliations:** 1 Department of Life Science, Gachon University, Sungnam, Gyeonggido 13120, Republic of Korea; 2 Lipidomia Inc., Sungnam, Gyeonggido 13120, Republic of Korea

**Keywords:** Puromycin, antibiotics, puromycin *N*-acetyltransferase, cell penetrating peptide, transduction

## Abstract

Puromycin N-acetyltransferase (PAC) is an enzyme that catalyzes the acetylation of puromycin, an inhibitor of protein synthesis. The PAC gene is often co-transfected with genes of interest in the same vector to serve as a selective marker, conferring puromycin resistance to mammalian cells. Cell-penetrating peptides (CPPs), which are 5-30 amino acids in length, facilitate the translocation of functional cargoes across the cell membrane. Among these, the HIV-transactivator of transcription (TAT) sequence is widely applied for its cell-penetrating and protein-delivery capabilities. In this study, we investigated whether attachment of the TAT sequence to PAC (TAT-PAC) enables intracellular delivery of TAT-PAC protein into mammalian cells, thereby conferring puromycin resistance. A recombinant TAT-PAC protein was expressed in *Escherichia coli* and purified to homogeneity. The purified TAT-PAC protein retained enzymatic activity, with a specific activity of 197 nmol/min/mg. Intracellular delivery of TAT-PAC was confirmed using confocal microscopy and flow cytometry, employing an RFP (red fluorescent protein)-tagged TAT-PAC fusion protein. Treatment of HEK293 and SY5Y cells with TAT-PAC resulted in increased cell viability in the presence of puromycin, demonstrating its functionality as a selection marker. This study suggests the potential application of cell-permeable PAC protein for selection of co-delivered therapeutic or gene-editing proteins in mammalian cells, providing a promising alternative to traditional genetic selection methods.

## Introduction

Antibiotic selective markers are essential tools for identifying and isolating cells that have successfully incorporated foreign genes through transformation or transfection [1, 2]. By introducing resistance-conferring genes, researchers can selectively propagate transformed cells while eliminating non-transformed counterparts. Common antibiotic-selective markers include neomycin, hygromycin B, blasticidin S, and puromycin resistance genes [3-5]. Each marker provides resistance to a specific antibiotic, allowing for selective growth in the corresponding antibiotic-containing medium.

Puromycin, an aminonucleoside antibiotic, inhibits protein synthesis in both prokaryotic and eukaryotic cells. It achieves this by mimicking the 3' end of aminoacyl-tRNA and occupying the A-site of the ribosome during translation [6]. Once incorporated, puromycin forms a peptide bond with the nascent polypeptide chain, releasing an incomplete polypeptide and prematurely terminating translation [7, 8]. This results in truncated, nonfunctional proteins and ultimately induces cell death [8].

**Fig. 1:**
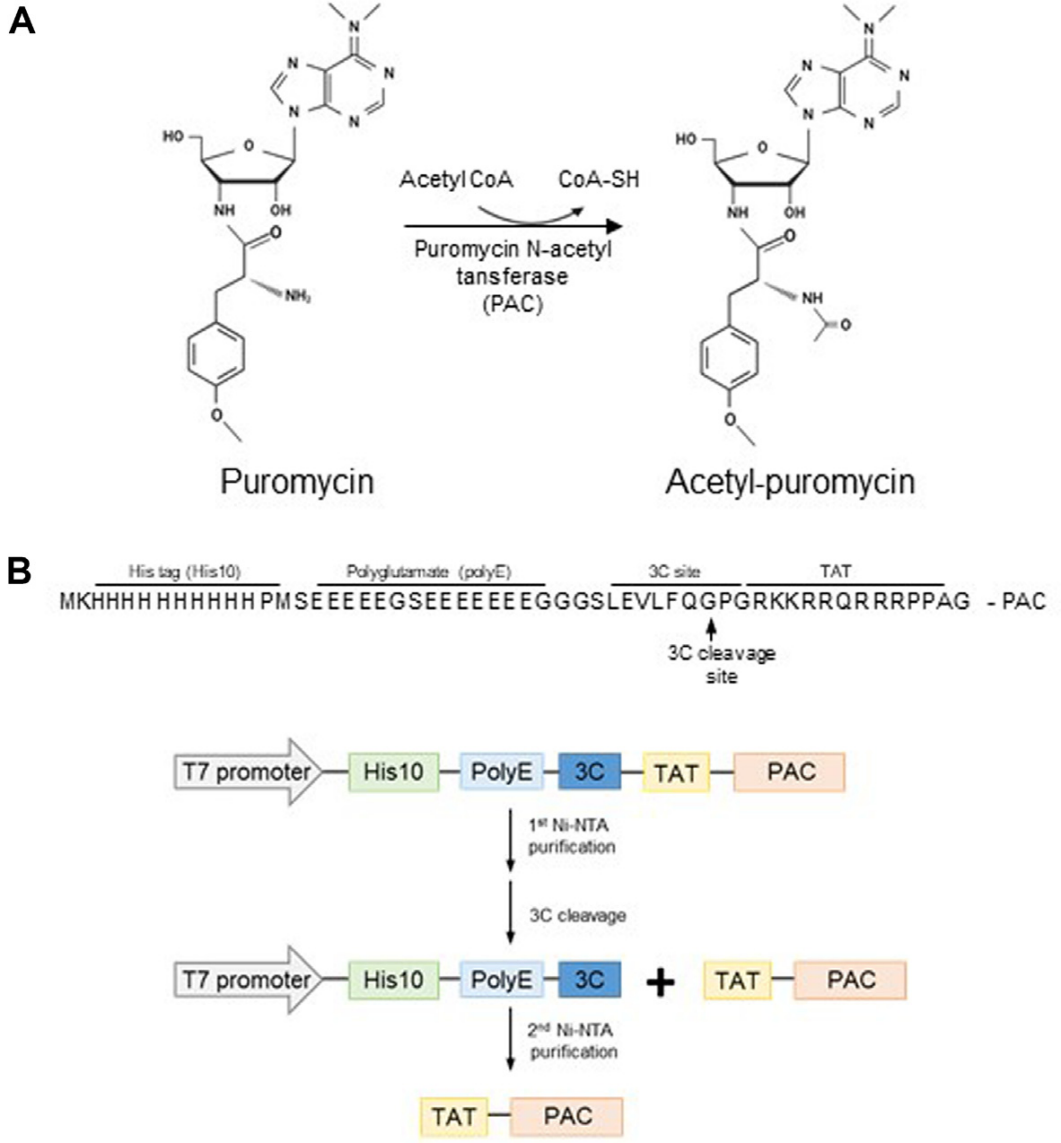
Puromycin *N*-acetyltransferase reaction (A) and the experimental scheme for recombinant TAT-PAC protein expression and purification (B).

Puromycin resistance is conferred by puromycin N-acetyltransferase (PAC), a bacterial enzyme that inactivates puromycin through *N*-acetylation of its reactive amino group (Fig. 1A). PAC was initially isolated from *Streptomyces alboniger* and first employed as a selection marker for transfected mammalian cells in 1988 [9, 10]. Cells expressing PAC can survive and proliferate in the presence of puromycin, making it a widely used tool in molecular biology for gene expression studies and the generation of stable cell lines [11]. Despite its utility, the conventional puromycin selection method has limitations. Plasmid-based delivery of PAC may result in random genomic integration, potentially disrupting essential genes or regulatory elements. Moreover, not all cells take up the plasmid, leading to a heterogeneous population (mosaicism) that necessitates additional selection and validation steps. These limitations highlight the need for alternative strategies to improve puromycin selection efficiency.

Cell-penetrating peptides (CPP) offer a promising solution to these challenges. CPP are short peptides, typically 5-30 amino acids in length, that can efficiently traverse cell membranes and deliver diverse cargoes—such as proteins, nucleic acids, and nanoparticles—into cells through energy-dependent and independent pathways [12]. CPP are often cationic, with positively charged residues like arginine and lysine facilitating interactions with negatively charged cell membranes [13]. Unlike traditional delivery methods, CPP enable non-invasive cellular entry without compromising membrane integrity, making them a safe and efficient cargo delivery strategy [14].

Among CPPs, the trans-activator of transcription (TAT) peptide, derived from the human immunodeficiency virus type 1 (HIV-1), is one of the most extensively studied [15]. TAT is a cationic protein comprising 86-101 amino acids, depending on the viral strain [16, 17]. Its basic domain (residues 48-60), rich in arginine and lysine residues, is responsible for its cell-penetrating properties [18, 19]. This domain interacts with negatively charged cell membrane components such as glycosaminoglycans and phospholipids, facilitating cellular uptake through endocytosis or direct translocation [19]. These properties make TAT a versatile tool for intracellular protein delivery in biotechnology and drug delivery research.

In this study, we aimed to exploit the cell-penetrating properties of the TAT peptide to enable the intracellular delivery of PAC protein, thereby enhancing puromycin selection efficiency. By fusing TAT to PAC, we sought to facilitate direct translocation of the enzyme across the cell membrane, replacing the need for plasmid-based vectors or complex delivery systems. This approach simplifies experimental workflows and improves the specificity and efficiency of puromycin-based selection. Our findings provide a novel strategy for protein delivery and a robust tool for molecular biology research, offering alternative method for selecting cells expressing genes of interest and advancing gene therapy and cell engineering studies.

## Materials and Methods

### Construction of Plasmid and Expression Condition

The PAC-coding gene was amplified from pBABEpuro_myc_ER (Addgene, #19128), and the plasmid backbone was amplified from pETM11. The vector was cloned using multiple PCR steps. Sequence- and ligation-independent cloning (SLIC) was used to insert polyglutamate (polyE) and an HRV 3C cleavage site into pETm11-10His-TAT-LS [20-22]. The plasmid backbone was amplified by PCR using the following primers: forward, 5'-GAAGTTTTGTTTCAAGGTCCAGGTCGCAAGAAACGTCGC-3'; reverse, 5'-GAACCCTCTTCTTCCTCT TCGCTCATGGGGTGATGGTGG-3'. The resulting PCR fragments were treated with DpnI (New England Biolabs, USA) at 37°C for 1 h and purified using a gel purification kit (Bionics, Republic of Korea). To generate an overhang, an exonuclease reaction was performed using T4 DNA polymerase. Specifically, 200 ng of the vector PCR product was treated with 0.25 U T4 DNA polymerase (New England Biolabs) at 22°C for 30 min, and the reaction was stopped by adding 1/10 volume of 10 mM dCTP. Insertion of polyglutamate and the HRV 3C site was achieved through primer annealing. The annealing reaction contained 50 mM Tris-HCl (pH 8.0), 200 mM NaCl, and 4.5 μM of each primer: forward, 5'-GAAGAGGAAGAAGAGGGTTCAGAAGAAGAAGAGGAAGAGGAGGGC GGTGGTTCCTTAG-3'; reverse, 5'-TGGACCTTGAAACAAAACTTCTAAGGAACCACCGCCCTCCTCTTC CTCTTCTTCTTCT-3'. The reaction mixture was heated at 90°C for 3 min and then cooled to room temperature. The annealed fragment was purified via ethanol precipitation. Both PCR fragments were ligated at a 1:1 molar ratio using T4 DNA ligase (New England Biolabs) at 37°C for 30 min, and the ligation product was immediately transformed into DH5α competent *E. coli* cells. The cloned plasmid was verified through restriction enzyme digestion and sequencing. As a negative control, the TAT sequence was removed from the expression vector of TAT-PAC to generate PAC-only protein. To validate transduction of PAC and TAT-PAC, a red fluorescent protein (RFP) tag was inserted at the C-terminus of the PAC coding gene using PCR-based Gibson assembly.

For protein expression, 1 μl of the previously cloned plasmid was transfected into 100 μl of BL21(DE3) competent *E. coli* cells. After transformation, cells were cultured in 1 L of Luria-Bertani (LB) broth at 37°C until OD600 reached 0.6-0.8. The culture was then incubated at 4°C for at least 30 min while the incubator was cooled to 16°C. Expression of recombinant proteins was induced with 0.5 mM isopropyl β-D-1-thiogalactopyranoside (IPTG) at 16°C overnight. Cells were harvested by centrifugation at 6,000 rpm for 30 min and either used directly for further purification or stored at -80°C until lysis.

### Affinity Purification by Ni-NTA Column Chromatography

*E. coli* cells transformed with the TAT-PAC-expressing plasmid were lysed by ultrasonication in 20 ml of Ni-binding buffer (20 mM Tris-HCl, pH 8.0, 500 mM NaCl, 0.05% Tween 20, 5 mM β-mercaptoethanol, 5% glycerol) for 40 min. Cell debris was removed by centrifugation at 16,000 rpm for 30 min. The supernatant was filtered using a 0.8 μm syringe filter (ADVANTEC, Taiwan) and loaded onto a 1 ml Ni-NTA column pre-equilibrated with Ni-binding buffer. The column was washed sequentially with Ni-binding buffer containing 5 mM imidazole, followed by 30 mM imidazole, and the protein was eluted using elution buffer (Ni-binding buffer with 250 mM imidazole). The eluted protein was dialyzed in HRV 3C cleavage buffer (50 mM Tris-HCl, pH 8.0, 150 mM NaCl, 10 mM MgCl_2_, 0.1% Tween 20, 1 mM DTT, 0.75 M trehalose) at 4°C. The dialyzed protein was digested with 3C protease at 4°C for 16 h, followed by a second dialysis in Ni-binding buffer. The digested protein solution was loaded onto a 1 ml Ni-NTA column pre-equilibrated with Ni-binding buffer. The flow-through contained the purified His tag-free TAT-PAC, which was subsequently dialyzed in storage buffer (20 mM Tris-HCl, pH 8.0, 150 mM NaCl, 5 mM β-mercaptoethanol, 0.1 mM EDTA, 50% glycerol) and stored at -80°C for further characterization. Each step of the purification process was confirmed by SDS-PAGE using a 12% gel, followed by Coomassie blue staining.

### *In vitro* TAT-PAC Enzyme Assay

The enzyme activity of TAT-PAC was measured by conducting an acetylation reaction of puromycin and quantifying the absorbance of the colorimetric product in a 96-well plate. The reaction was initiated by adding 100 μl of diluted TAT-PAC enzyme and 100 μl of reaction buffer containing 0.4 mM acetyl-CoA, 0.4 mM puromycin, 0.4 mM 5,5'-dithiobis(2-nitrobenzoic acid) (DTNB) (Sigma, USA) [23], and 2 mg/ml bovine serum albumin (BSA) in Ni-binding buffer. The reaction proceeded for 10 min at room temperature, after which the absorbance at 412 nm was measured using a spectrophotometer. TAT-PAC enzyme activity was normalized against a standard curve generated using cysteine as a sulfhydryl group donor [9, 24]. To determine enzyme activity at various substrate concentrations, 5 μg/ml of TAT-PAC was used. As a negative control, TAT-PAC protein was inactivated by incubation at 100°C for 30 min and the enzyme activity was measured as described above.

### Cell Viability Assay

SY5Y and HEK293 cells were obtained from ATCC (American Type Culture Collection, USA). These cells were cultured in Dulbecco's Modified Eagle Medium (DMEM, Welgene) supplemented with 10% (v/v) Fetal Bovine Serum (FBS) and 1% (v/v) Penicillin-Streptomycin (Welgene, Republic of Korea) in a humidified incubator at 37°C and 5% CO_2_. On the day prior to TAT-PAC treatment, 5 × 10^4^ cells were seeded into a 96-well plate in culture medium. The protein was administered three times at 4-h intervals. Various concentrations of TAT-PAC (100 μl per well) were added to the culture medium. Following treatment, 100 μl of puromycin, at optimal concentrations for each cell line, was introduced to the medium, and cells were incubated for 24, 48, and 72 h. The optimal puromycin concentration for each cell line was determined by assessing cell viability at 72 h using CCK-8 assay (R&D Systems, USA) by colorimetric measurement at 450 nm according to the manufacturer’s instruction.

Alternatively, the pCDH-3xFLAG-GFP-puroR plasmid (Addgene #167463), which contains the PAC gene, was transfected into HEK293 or SY5Y cells using the Lipidofect P transfection reagent, following the manufacturer’s instructions (Republic of Korea). Cell viability was then measured using CCK-8 assay.

### Transduction of RFP Tagged TAT-PAC Inside Cells

Transduction of TAT-PAC was confirmed through confocal microscopic imaging of RFP-tagged PAC in HEK293 or SY5Y cells respectively. One day prior to protein treatment, 5 × 10^5^ cells were seeded into culture plates. TAT-PAC-RFP treatment was administered at two different protein concentrations (1 μg/ml and 2.5 μg/ml) in MEM medium. After a 4-h incubation with the protein, cells were treated with 1 μg/ml Hoechst stain for 15 min in a humidified incubator at 37°C and 5% CO_2_. Fluorescence was observed using a Nikon Eclipse C1 confocal laser scanning microscope. PAC-RFP was used as a negative control.

### Cell Transduction Efficiency of TAT-PAC

For the analysis of transduction efficiency, 1 × 10^6^ HEK293 or SY5Y cells were seeded onto each well of a 6-well plate and allowed to adhere overnight. The cells were treated with purified TAT-PAC-RFP at various concentrations by diluting the protein in culture medium for 4 h. After treatment, the cells were detached using trypsin, collected by centrifugation, and resuspended in 1 ml PBS. The cell suspension was passed through a cell strainer to remove cell clumps. Following filtration, the cells were analyzed for transduction efficiency using a Cytomics FC500 MLP cytometer (Beckman Coulter).

## Results

### Expression and Purification of TAT-PAC in *E. coli*

**Fig. 2:**
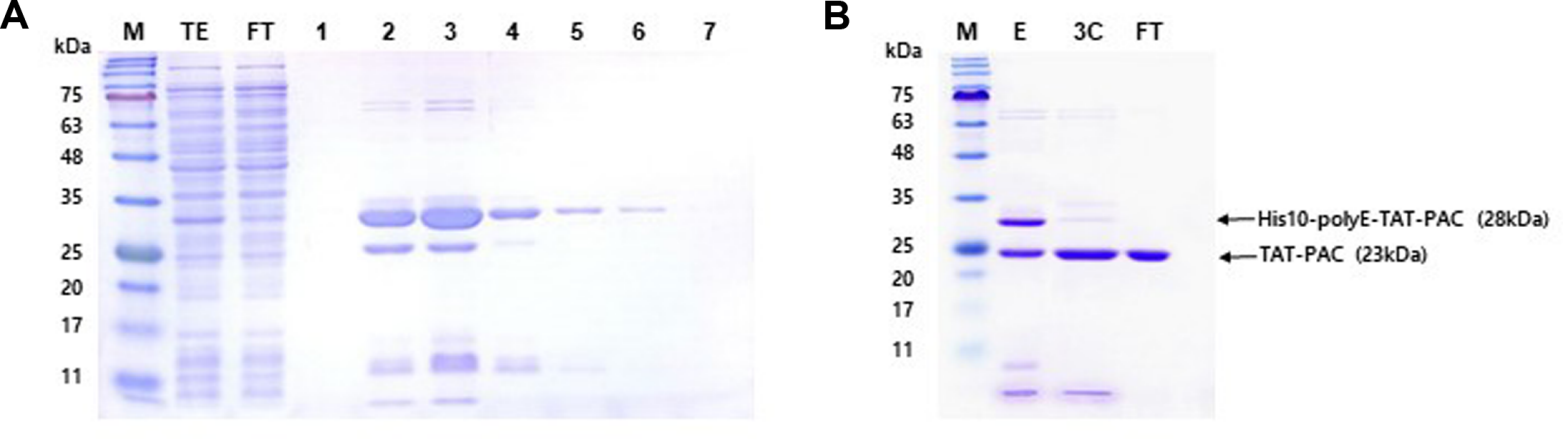
Purification of TAT-PAC. (**A**) First purification of His10-polyE-TAT-PAC using Ni-NTA affinity chromatography. The total extract (TE) was obtained from *E. coli* transformed with the His10-polyE-TAT-PAC plasmid via sonication. The first flow-through (FT) contained proteins lacking the His tag, whereas proteins with a His tag, including His10-polyE-TAT-PAC, bound to the Ni-NTA resin. The column was washed sequentially with Ni-binding buffer containing 5 mM imidazole and 30 mM imidazole. His10-polyE-TAT-PAC (28 kDa) was eluted with elution buffer containing 250 mM imidazole, and the collected fractions (1 ml, fractions 1-7) were analyzed by SDS-PAGE and visualized using Coomassie blue staining. (**B**) Second purification of TAT-PAC was performed by pooling the eluted fractions (**E**). His10-polyE-TAT-PAC was digested with 3C protease and passed through the Ni-NTA column, yielding TAT-PAC lacking His10 and polyE tags in the second flow-through (FT). M indicates molecular weight markers.

The PAC-coding gene was inserted into the expression vector pETm11 according to the scheme in Figure 1B. A polyglutamate (polyE) sequence was inserted to neutralize the positive charges from TAT, which is directly linked to PAC [20]. Expression of PAC Protein was induced using IPTG, and the bacterial culture was harvested by centrifugation, followed by cell lysis via sonication. The supernatant, containing the TAT-PAC protein, was collected and subjected to purification using Ni-NTA affinity chromatography. During the initial purification step, the majority of TAT-PAC was eluted in fractions 2-5 (Fig. 2A). The pooled TAT-PAC fractions were subsequently cleaved using HRV 3C protease. Prior to cleavage, TAT-PAC, which includes a His10 tag and a 3C protease cleavage site, exhibited a molecular weight of 28 kDa. Following 3C protease cleavage, the processed TAT-PAC had a molecular weight of 23 kDa (Fig. 2B). Since the cleaved TAT-PAC lacks the His10 tag, it did not bind to the Ni-NTA resin. To remove residual His-tagged contaminants, a negative selection step was performed using Ni-NTA chromatography, during which the flow-through fraction containing TAT-PAC was collected. Proteins retaining the His10 tag remained bound to the Ni-NTA resin and were thereby eliminated. Additionally, TAT-PAC-RFP was purified using the same procedures as TAT-PAC (data not shown).

### Measurement of Activity of TAT-PAC in Cell-Free System

**Fig. 3:**
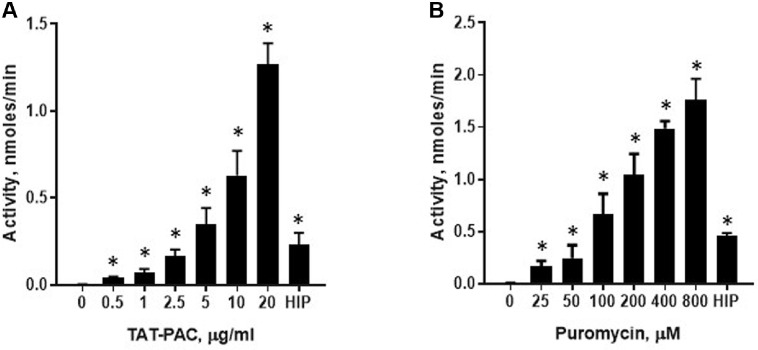
Measurement of TAT-PAC enzyme activity in a cell-free system. Enzyme activity was assessed at various TAT-PAC protein concentrations (**A**) and puromycin concentrations (**B**). For enzyme concentration-dependent activity, 0.2 mM puromycin was used, while 5 μg/ml of TAT-PAC was used for substrate-dependent activity. Colorimetric absorbance was measured at 412 nm. HIP, heat-inactivated TAT-PAC at 100°C for 30 min. Data are presented as mean ± SEM (*n* = 3). *p < 0.05 vs. no-enzyme or no-substrate control.

TAT-PAC activity was assessed using a coupled assay with colorimetric detection of CoA, which reacts with Ellman’s reagent (DTNB) during the acetylation of puromycin in a cell-free system. In this assay, puromycin is first acetylated by PAC using acetyl-CoA as the acetyl donor. The sulfhydryl group of the CoA produced during the enzymatic reaction subsequently reacts with DTNB, generating a colorimetric product (Fig. 1A). This product was quantified colorimetrically at 412 nm. To evaluate TAT-PAC activity, puromycin (200 μM) was incubated with varying concentrations of TAT-PAC for 20 min. Additionally, substrate-dependent activity was assessed by incubating TAT-PAC (5 μg/ml) with different concentrations of puromycin. Absorbance was recorded within the first 20 min, ensuring a linear response. Enzyme activity increased proportionally in substrate- and enzyme-dependent manner (Fig. 3A and 3B). The specific activity of TAT-PAC was determined to be 197 nmol/min/mg. We also measured the enzyme activity of heat-inactivated TAT-PAC (HIP) as a negative control, and it still exhibited approximately 20% of the maximal activity (Fig. 3). These results indicate that purified TAT-PAC exhibits enzymatic activity *in vitro*.

### Cellular localization of TAT-PAC-RFP

**Fig. 4:**
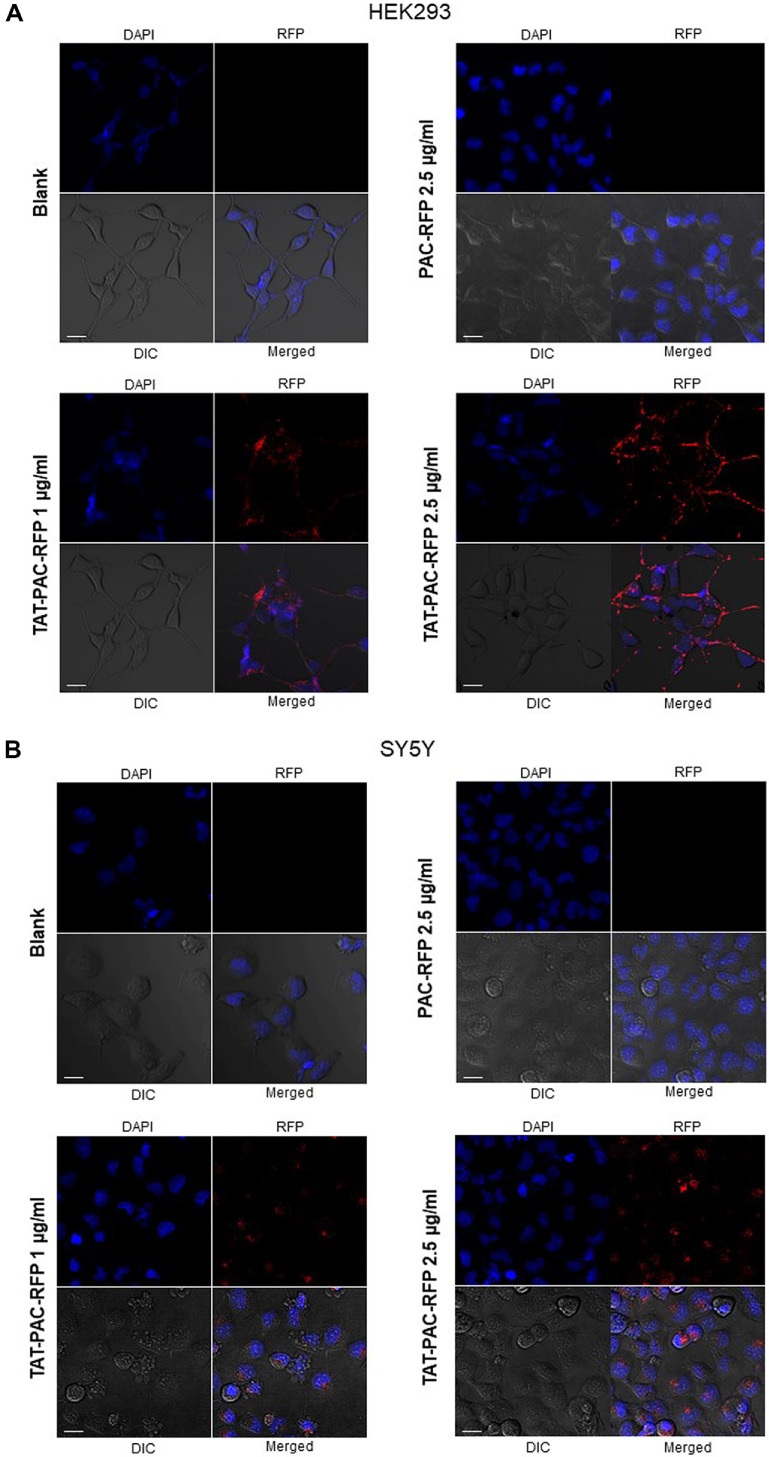
Localization of TAT-PAC-RFP in HEK293 (**A**) and SY5Y (**B**) cells. Cells were treated with 1 or 2.5 μg/ml TATPAC- RFP, or alternatively with 2.5 μg/ml PAC-RFP, for 4 h. Nuclei were stained with Hoechst (blue), and RFP fluorescence (red) was detected using confocal microscopy with an excitation peak at 590 nm and an emission peak at 612 nm. Scale bar = 20 μm.

To determine whether TAT-PAC is internalized into cells, its transduction efficiency was evaluated using confocal imaging analysis. To visualize TAT-PAC localization, red fluorescent protein (RFP) was fused to the C-terminus of TAT-PAC to prevent interference of transducing ability of CPP. HEK293 or SY5Y cells were treated with TAT-PAC-RFP at concentrations of 1 and 2.5 μg/ml for 4 h, after which the RFP signal was analyzed using confocal microscopy. The results showed that extracellular TAT-PAC-RFP was successfully translocated into the cytoplasm of both cell types. Furthermore, cells treated with 2.5 μg/ml TAT-PAC-RFP exhibited a higher intracellular RFP signal compared to those treated with the lower concentration (Fig. 4A and 4B). Additionally, TAT-PAC-RFP was localized in the cytoplasm, distinct from the Hoechst-stained nucleus. In contrast, PAC-RFP lacking the TAT sequence was not translocated into HEK293 or SY5Y cells. These findings indicate that TAT-PAC efficiently penetrates cells and accumulates in the cytoplasm in a dose-dependent manner.

**Fig. 5:**
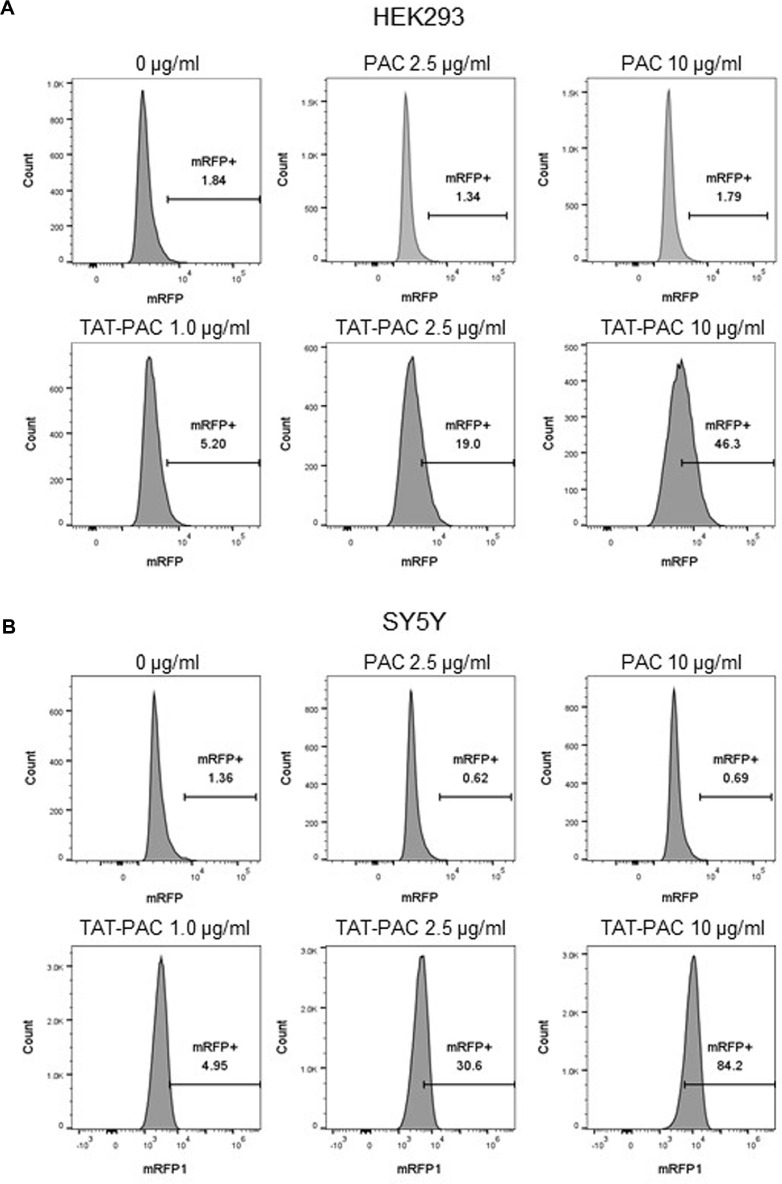
Flow cytometric analysis of transduction efficiency of TAT-PAC-RFP and PAC-RFP. HEK293 and SY5Y cells were treated with various concentrations of TAT-PAC-RFP or PAC-RFP for 4 h and analyzed using Cytomics FC500 MLP cytometer equipped with a 590 nm laser for excitation and 610 nm for emission. (**A**) Histogram of RFP fluorescence in HEK293 cells treated with different concentrations of TAT-PAC-RFP or PAC-RFP. (**B**) Histogram of RFP fluorescence in SY5Y cells treated with different concentrations of TAT-PAC-RFP or PAC-RFP.

To further assess the transduction efficiency of TAT-PAC, flow cytometry analysis was performed. Untreated control cells were used as a gating reference to exclude cell debris from the analysis of internalized TAT-PAC-RFP. In HEK293 cells treated with TAT-PAC-RFP for 4 h, 5.2% of total cells exhibited an RFP signal at 1 μg/ml, while 19% and 46.3% of cells showed RFP fluorescence at 2.5 μg/ml and 10 μg/ml, respectively (Fig. 5A). Similarly, in SY5Y cells, 4.95% of total cells displayed an RFP signal at 1 μg/ml, whereas 30.6% and 84.2% of cells exhibited fluorescence at 2.5 μg/ml and 10 μg/ml, respectively (Fig. 5B). Again, PAC-RFP lacking the TAT sequence was not translocated into HEK293 or SY5Y cells. These results demonstrate that TAT-PAC efficiently transduces into cells in a dose-dependent manner and localizes to the cytoplasm, suggesting its potential role in conferring puromycin resistance.

### TAT-PAC Activity in SY5Y and HEK293 Cells

To determine the optimal puromycin concentration for each cell line, cell viability was assessed in the presence of varying puromycin concentrations. SY5Y and HEK293 cells were plated in a 96-well plate with culture media one day before antibiotic treatment. The cells were then exposed to fresh media containing different concentrations of puromycin, and cell viability was measured after various times. In HEK293 cells, treatment with 1 μg/ml puromycin did not significantly affect cell viability; however, concentrations above 2 μg/ml reduced cell viability by 55% (data not shown). At 5 μg/ml, HEK293 cell viability decreased by 94%. In contrast, SY5Y cells exhibited a 90%reduction in viability at 2 μg/ml puromycin (data not shown). Based on these findings, puromycin concentrations of 2 μg/ml for HEK293 cells and 1.5 μg/ml for SY5Y cells were selected to assess the puromycin resistance conferred by TAT-PAC.

**Fig. 6:**
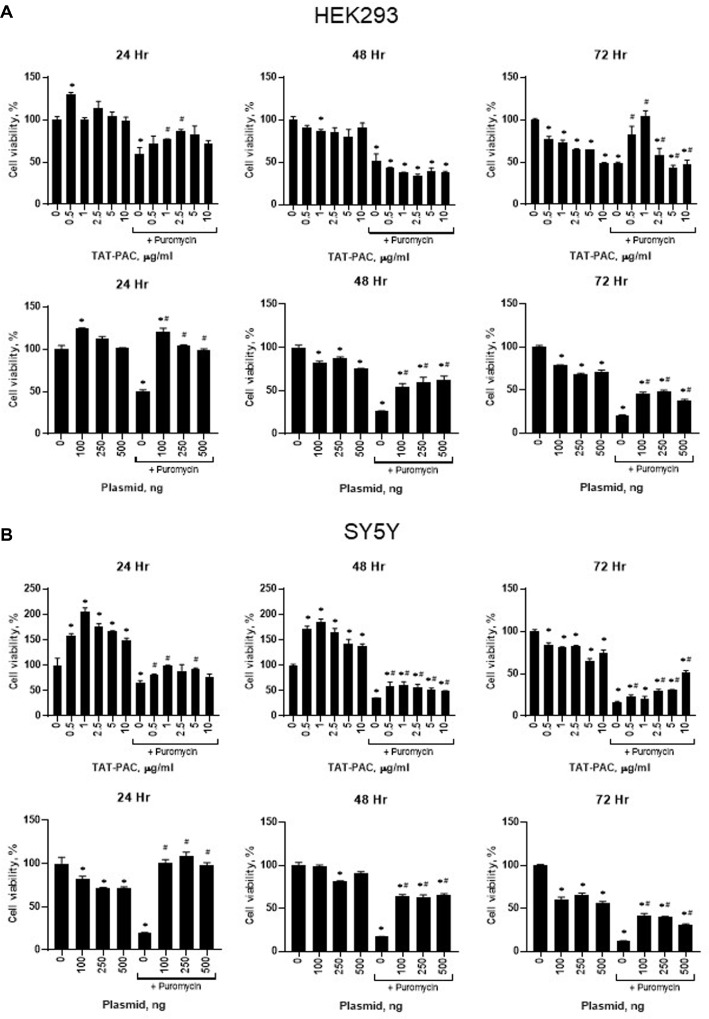
Restoration of cell viability by TAT-PAC in HEK293 and SY5Y cells treated with puromycin. HEK293 (**A**) and SY5Y (**B**) cells were treated with different concentrations of TAT-PAC every 24 h and incubated for 24, 48, and 72 h in HEK293 cells and and cell viability was assessed using the CCK-8 assay. Alternatively, HEK293 (**A**) and SY5Y (**B**) cells were transfected with pCDH-3xFLAG-GFP-puroR containing *pac* and incubated for 24, 48, and 72 h in HEK293 cells and cell viability was assessed using the CCK-8 assay. Data are presented as mean ± SEM, *n* = 6. *p < 0.05 vs. no-treatment control; #*p* < 0.05 vs. puromycin-only control.

Next, we investigated whether TAT-PAC treatment confers puromycin resistance in HEK293 and SY5Y cells. Cells were treated with TAT-PAC by directly adding it to the culture media. In HEK293 cells, puromycin treatment alone resulted in 59% cell viability compared to the no-treatment control. However, co-treatment with 2.5 μg/ml TAT-PAC in the presence of puromycin for 24 h significantly increased cell viability to 87% (Fig. 6A). At 48 h, no substantial difference in cell viability was observed between the puromycin-treated control and the TAT-PAC-treated group. However, after 72 h of TAT-PAC treatment, 1 μg/ml TAT-PAC restored cell viability to levels comparable to the no-treatment control, whereas cells treated with puromycin alone exhibited only 48% viability (Fig. 6A). Cell survival increased with TAT-PAC concentrations up to 1 μg/ml but declined at higher concentrations in HEK293 cells. Similarly, in SY5Y cells, treatment with 1 μg/ml TAT-PAC resulted in 98% cell viability in the presence of puromycin compared to the no-treatment control, while puromycin treatment alone resulted in 65%viability at 24 h (Fig. 6B). At 48 h, 1 μg/ml TAT-PAC maintained the highest cell viability among TAT-PAC-treated groups, with 60% viability compared to the no-treatment control, whereas puromycin-treated cells exhibited only 35% viability (Fig. 6B). By 72 h, SY5Y cells continued to show increased viability. Treatment with 10 μg/ml TAT-PAC resulted in 51% viability compared to the no-treatment control, while puromycin-treated cells exhibited only 16% viability (Fig. 6B). These findings suggest that TAT-PAC treatment confers puromycin resistance in both SY5Y and HEK293 cells, indicating its potential as an efficient method for selecting cells transduced with proteins of interest.

When HEK293 or SY5Y cells were transfected with a plasmid containing the *pac* gene, the decrease in cell viability caused by puromycin was restored to the level of the untreated control at 24 h post-transfection (Fig. 6A and 6B). Up to 72 h, puromycin resistance conferred by the *pac* gene increased cell viability at all DNA doses in the presence of puromycin, compared to the puromycin-only control. However, after 72 h, the level of cell survival was lower than that observed in TAT-PAC-transduced cells in both HEK293 and SY5Y cells. These results suggest that TAT-PAC transduction is more effective in conferring puromycin resistance and promoting cell survival than *pac* plasmid transfection at 72 h post-treatment, despite the high selection efficiency of plasmid-based methods.

## Discussion

Puromycin is an antibiotic that inhibits protein synthesis, thereby compromising the survival of eukaryotic cells. PAC inactivates puromycin, enabling the selection of transfected cells. However, the conventional puromycin selection method is limited by issues such as random plasmid integration and mosaicism in the genome. To address these limitations, we propose the attachment of TAT, a CPP derived from HIV-1, to facilitate the direct translocation of PAC across the cell membrane. In this study, we found the following: 1) TAT-PAC was successfully expressed and purified in *E. coli*, 2) the purified recombinant TAT-PAC retained intact enzyme activity in a cell-free system, 3) TAT-PAC was efficiently transduced into mammalian cells, and 4) TAT-PAC conferred resistance to puromycin in mammalian cells.

Purification of TAT-PAC was performed using a two-step Ni-NTA affinity chromatography procedures. In the first purification step, His10-polyE-TAT-PAC was eluted with a Ni-binding buffer containing 250 mM imidazole. The His10-polyE-TAT-PAC fusion protein was then cleaved into His10-polyE and TAT-PAC by 3C protease. His10-polyE was removed during a second round of Ni-NTA affinity chromatography, and TAT-PAC was obtained from the flow-through of this second purification step (Fig. 1B). Due to the presence of positively charged amino acids such as arginine (Arg) and lysine (Lys) in the TAT peptide, TAT-tagged proteins tend to accumulate in inclusion bodies [25]. We previously demonstrated that the addition of polyE to TAT-tagged proteins improves protein expression yield in *E. coli* [20]. Based on these findings, we incorporated polyE into the TAT-PAC construct. Furthermore, TAT-PAC was expressed at 16°C to prevent the formation of inclusion bodies, which often occur at 37°C, where the majority of TAT-PAC remains insoluble and inactive. Incubating at a lower temperature facilitated the production of properly folded and soluble TAT-PAC, as previously reported [26, 27]. During 3C protease cleavage, we observed that TAT-PAC tended to aggregate and precipitate. As previously noted, the addition of trehalose could solubilize proteins and prevent precipitation [28, 29]. Consequently, we optimized the expression and purification procedures for TAT-PAC by incorporating the polyE domain, incubating at low temperature, and adding trehalose to the 3C cleavage buffer.

The purified TAT-PAC enzyme was characterized using an *in vitro* enzyme assay. TAT-PAC activity was measured by a coupled-assay method utilizing the DTNB compound, which quantifies free sulfhydryl groups in solution that react with the sulfhydryl group of CoA, a product of the enzyme reaction [9, 24]. Both protein-dependent and substrate-dependent activities increased in a concentration-dependent manner (Fig. 3A and 3B). Rapoport *et al*. demonstrated that the addition of TAT did not affect the protein function and could be utilized for the endocytosis of therapeutic proteins [30]. In agreement with this, we found that the protein activity of recombinant TAT-PAC remained intact and was not affected by the addition of the TAT domain [31, 32].

We observed that RFP-tagged TAT-PAC was localized inside the cells, particularly in the cytoplasm, when HEK293 and SY5Y cells were treated with TAT-PAC in the media (Fig. 4). Consistent with the confocal imaging results, the proportion of cells exhibiting the RFP signal increased in a TAT-PAC concentration-dependent manner (Fig. 5). In our previous study, the attachment of a nuclear localization signal (NLS) to Cre facilitated its delivery to the nucleus [20]. However, PAC is known to localize and function in the cytoplasm when cells are transfected with a vector expressing the PAC gene [33]. Consequently, in this study, we used only the TAT peptide for the delivery of PAC to the cytoplasm. In addition to the ability of TAT to penetrate the cell membrane, other cell-penetrating peptides (CPPs) can deliver cargo to specific organelles, including the nucleus, mitochondria, and lysosomes [34-36]. Depending on the mechanism of action and the intended target, various delivery domains can be employed for gene modification and therapeutic applications.

Cell viability was assessed to evaluate TAT-PAC activity in SY5Y and HEK293 cells and to determine the optimal concentration of TAT-PAC for selecting transduced cells in the presence of puromycin. The optimal puromycin concentrations for TAT-PAC-mediated antibiotic resistance in SY5Y and HEK293 cells were determined by monitoring significant changes in cell viability following puromycin treatment. In SY5Y cells, cell viability increased with increasing concentrations of TAT-PAC protein, whereas HEK293 cells exhibited the highest viability at 1 μg/ml TAT-PAC. While no cytotoxicity was observed in SY5Y cells, HEK293 cells showed a slight decrease in cell proliferation at higher TAT-PAC concentrations after 72 h. When cells were transfected with a plasmid containing the *pac* gene, a similar decrease in cell viability was observed (Fig. 6), which may be attributed to bacterial PAC expression and differential responses among mammalian cell types. TAT-PAC demonstrated variable effects on puromycin resistance and cell viability depending on the concentration and cell type [24, 37, 38]. For example, the optimal puromycin concentration for selecting transduced cells was reported to be 4 μg/ml in RW4 mouse embryonic stem cells [39], while 2.5 μg/ml was found to be optimal in KH2 ES cells [24, 37, 38, 40]. Since purified TAT-PAC is designed for use in protein co-transduction assays, it has potential for broad application across various cell lines [41]. However, the optimal concentration should be determined based on the specific cell types and this deserves further investigation.

When cells were transfected with a plasmid containing *pac*, puromycin resistance was observed 24 h post-transfection, and a clear distinction was evident between transfected and non-transfected controls in the presence of puromycin (Fig. 6). Compared to TAT-PAC protein transduction, plasmid transfection was more effective at selecting transfected cells within a short period, likely due to the consistently high expression of PAC from the plasmid. Notably, 72 h post-transduction with TAT-PAC, cell survival was higher in TAT-PAC-treated cells than in pac-transfected cells. When using Cas9 or Cas12 proteins for gene editing, a selection marker is typically not available. In this context, CPP-tagged TAT-PAC offers a distinct advantage over plasmid-based methods, as it enables direct protein delivery into cells without the need for DNA transfection.

Recent findings suggest that cell-penetrating Cas proteins, such as Cas9 or Cas12a, can achieve robust gene-editing efficacy when co-administered with a cell-penetrating endosomal escape peptide, TAT-HA2 [42]. Co-incubation with TAT-HA2, which is composed of the TAT sequence and an endosomal escape-facilitating segment derived from the influenza A virus hemagglutinin protein (HA2), significantly enhances the cellular uptake and nuclear localization of cell-penetrating Cas9. Whether protein transduction using such penetration enhancers improves the overall permeability and functional delivery of cell-penetrating proteins such as TAT-PAC warrants further investigation.

Collectively, the purification and characterization of cell-permeable TAT-PAC have provided valuable insights into its potential applications in cellular studies. The successful purification of the enzyme has enabled the determination of its kinetic parameters and substrate specificity, further advancing our understanding of its role as a selection markers. The characterization of the cell-permeable puromycin *N*-acetyltransferase provides a valuable tool for basic and applied research on protein transduction.

## References

[1] Browne SM, Al-Rubeai M (2007). Selection methods for high-producing mammalian cell lines. Trends Biotechnol..

[2] Mortensen R, Chestnut JD, Hoeffler JP, Kingston RE. 1997. Selection of transfected mammalian cells. *Curr. Protoc. Neurosci.* 4.6. 1-4.6. 20. 10.1002/0471142301.ns0406s00 18428489

[3] Kochupurakkal BS, Iglehart JD (2013). Nourseothricin N-acetyl transferase: a positive selection marker for mammalian cells. PLoS One.

[4] Gonzalez A, Jimenez A, Vazquez D, Davies J, Schindler D (1978). Studies on the mode of action of hygromycin B, an inhibitor of translocation in eukaryotes. Biochim. Biophys. Acta.

[5] Izumi M, Miyazawa H, Kamakura T, Yamaguchi I, Endo T, Hanaoka F (1991). Blasticidin S-resistance gene (bsr): a novel selectable marker for mammalian cells. Exper. Cell Res..

[6] Yarmolinsky MB, Haba GLDL (1959). Inhibition by puromycin of amino acid incorporation into protein. Proc. Natl. Acad. Sci. USA.

[7] Aviner R (2020). The science of puromycin: from studies of ribosome function to applications in biotechnology. Comput. Struct. Biotechnol. J..

[8] Nathans D (1964). Puromycin inhibition of protein synthesis: incorporation of puromycin into peptide chains. Proc. Natl. Acad. Sci. USA.

[9] Vara J, Malpartida F, Hopwood DA, Jiménez A (1985). Cloning and expression of a puromycin *N*-acetyl transferase gene from *Streptomyces alboniger* in Streptomyces lividans and *Escherichia coli*. Gene.

[10] Paik S-Y, Sugiyama M, Nomi R (1985). Isolation and properties of a puromycin acetyltransferase from puromycin-producing *Streptomyces alboniger*. J. Antibiot..

[11] De La Luna S, Ortín J (1992). pac Gene as efficient dominant marker and reporter gene in mammalian cells. Methods Enzymol..

[12] Lindgren M, Hällbrink M, Prochiantz A, Langel Ü (2000). Cell-penetrating peptides. Trends Pharmacol. Sci..

[13] Bechara C, Sagan S (2013). Cell-penetrating peptides: 20 years later, where do we stand?. FEBS Lett..

[14] Ter-Avetisyan G, Tunnemann G, Nowak D, Nitschke M, Herrmann A, Drab M (2009). Cell entry of arginine-rich peptides is independent of endocytosis. J. Biol. Chem..

[15] Green M, Ishino M, Loewenstein PM (1989). Mutational analysis of HIV-1 Tat minimal domain peptides: identification of transdominant mutants that suppress HIV-LTR-driven gene expression. Cell.

[16] Vogel BE, Lee SJ, Hildebrand A, Craig W, Pierschbacher MD, Wong-Staal F (1993). A novel integrin specificity exemplified by binding of the alpha v beta 5 integrin to the basic domain of the HIV Tat protein and vitronectin. J. Cell Biol..

[17] Ruben S, Perkins A, Purcell R, Joung K, Sia R, Burghoff R (1989). Structural and functional characterization of human immunodeficiency virus tat protein. J. Virol..

[18] Palmer T, Berks BC (2012). The twin-arginine translocation (Tat) protein export pathway. Nat. Rev. Microbiology.

[19] Erazo-Oliveras A, Muthukrishnan N, Baker R, Wang T-Y, Pellois J-P (2012). Improving the endosomal escape of cell-penetrating peptides and their cargos: strategies and challenges. Pharmaceuticals.

[20] Kim AH, Lee S, Jeon S, Kim GT, Lee EJ, Kim D (2020). Addition of an N-terminal poly-glutamate fusion tag improves solubility and production of recombinant TAT-Cre recombinase in *Escherichia coli*. J. Microbiol. Biotechnol..

[21] Li MZ, Elledge SJ (2012). SLIC: a method for sequence-and ligation-independent cloning. Methods Mol. Biol..

[22] Li MZ, Elledge SJ (2007). Harnessing homologous recombination in vitro to generate recombinant DNA via SLIC. Nat. Methods.

[23] Habeeb A (1972). [37] Reaction of protein sulfhydryl groups with Ellman's reagent. Methods Enzymol..

[24] Caputo AT, Eder OM, Bereznakova H, Pothuis H, Ardevol A, Newman J (2021). Structure-guided selection of puromycin *N*-acetyltransferase mutants with enhanced selection stringency for deriving mammalian cell lines expressing recombinant proteins. Sci. Rep..

[25] Lin Q, Jo D, Gebre-Amlak KD, Ruley HE (2004). Enhanced cell-permeant Cre protein for site-specific recombination in cultured cells. BMC Biotechnol..

[26] Francis DM, Page R. 2010. Strategies to optimize protein expression in *E. coli*. *Curr. Protoc. Protein Sci.* **61:** 5.24. 1-5.24. 29. 10.1002/0471140864.ps0524s61 20814932 PMC7162232

[27] Sahdev S, Khattar SK, Saini KS (2008). Production of active eukaryotic proteins through bacterial expression systems: a review of the existing biotechnology strategies. Mol. Cell. Biochem..

[28] Leibly DJ, Nguyen TN, Kao LT, Hewitt SN, Barrett LK, Van Voorhis WC (2012). Stabilizing additives added during cell lysis aid in the solubilization of recombinant proteins. PLoS One.

[29] Jain NK, Roy I (2009). Effect of trehalose on protein structure. Protein Sci..

[30] Rapoport M, Lorberboum-Galski H (2009). TAT-based drug delivery system-new directions in protein delivery for new hopes?. Expert Opin. Drug Deliv..

[31] Clark E, Nava B, Caputi M (2017). Tat is a multifunctional viral protein that modulates cellular gene expression and functions. Oncotarget.

[32] Lichtenstein M, Zabit S, Hauser N, Farouz S, Melloul O, Hirbawi J (2021). TAT for enzyme/protein delivery to restore or destroy cell activity in human diseases. Life.

[33] Azzam M, Algranati I (1973). Mechanism of puromycin action: fate of ribosomes after release of nascent protein chains from polysomes. Proc. Natl. Acad. Sci. USA.

[34] Vasconcelos L, Pärn K, Langel Ü (2013). Therapeutic potential of cell-penetrating peptides. Ther. Deliv..

[35] Geng J, Wang J, Wang H (2023). Emerging landscape of cell-penetrating peptide-mediated organelle restoration and replacement. ACS Pharmacol. Transl. Sci..

[36] Horton KL, Stewart KM, Fonseca SB, Guo Q, Kelley SO (2008). Mitochondria-penetrating peptides. Chem. Biol..

[37] Fordjour FK, Owiredu S, Muendlein H, Plange J, Morrell J, Han J (2016). Creation of CD63-deficient HEK293 cell lines using a polycistronic CAS9/EGFP/HSVtk/PuroR expression vector. Matters.

[38] Kopera HC, Hilgarth RS, Kopas TL, Lanigan TM (2020). Development and validation of a reporter cell line for rapid AAV quality control assessment. MethodsX.

[39] Pardieck J, Harb M, Sakiyama-Elbert SE (2022). A transgenic mouse embryonic stem cell line for puromycin selection of V0V interneurons from heterogenous induced cultures. Stem Cell Res. Ther..

[40] Ni P, Zhang Q, Chen H, Chen L (2014). Inactivation of an integrated antibiotic resistance gene in mammalian cells to re-enable antibiotic selection. Biotechniques.

[41] Iwamoto M, Mori C, Hiraoka Y, Haraguchi T (2014). Puromycin resistance gene as an effective selection marker for ciliate Tetrahymena. Gene.

[42] Zhang Z, Baxter AE, Ren D, Qin K, Chen Z, Collins SM (2024). Efficient engineering of human and mouse primary cells using peptide-assisted genome editing. Nat. Biotechnol..

